# Supplementary material for: Promotion of growth factor signaling as a critical function of β-catenin during HCC progression
Source: Nat Commun. 2019 Apr 23;10:1909. doi: 10.1038/s41467-019-09780-z (PMC6478918; doi:10.1038/s41467-019-09780-z)
Supplement: Supplementary file 1 — Supplementary Information [file 41467_2019_9780_MOESM1_ESM.pdf]

SUPPLEMENTARY INFORMATION

Promotion of growth factor signaling as a critical function of β-catenin during HCC progression

Kim et al.

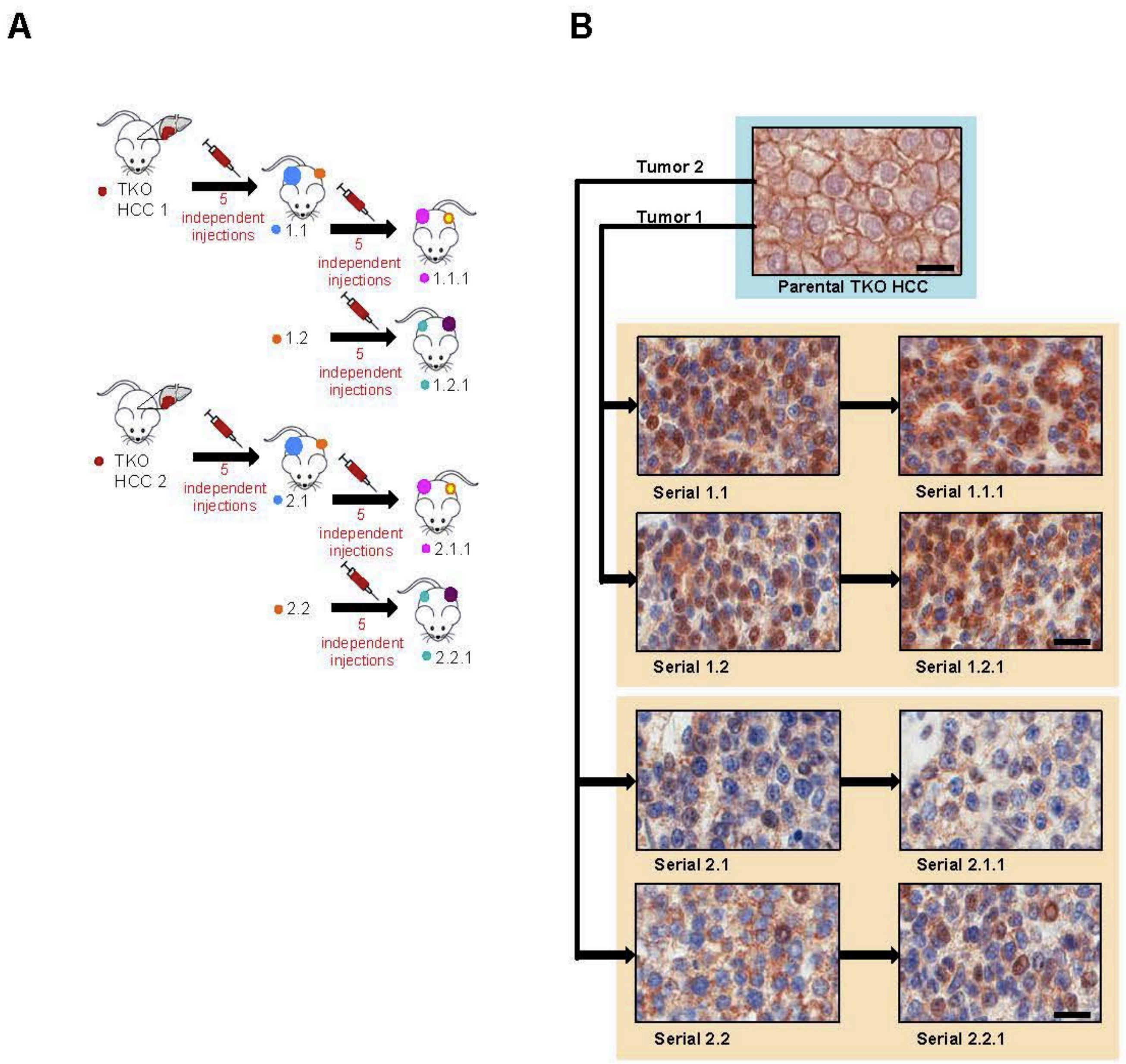

Supplementary Figure 1. Serial transplantation of TKO HCC

A Experimental design for the serial transplantation of TKO HCC. Numbers indicate the filiation of individual tumors. **B** Representative immunohistochemistry (IHC) for β-catenin in serially-transplanted subcutaneous tumors (Serial), showing clonal evolution and advanced tumor grade compared to primary tumors (TKO HCC). Scale bar=20μm.

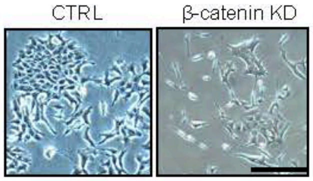

Supplementary Figure 2. TKO HCC cells upon loss of β-catenin

Representative phase contrast images of TKO HCC cells expressing control (CTRL) or β-catenin KD by shβcat3. Scale bar=250μm.

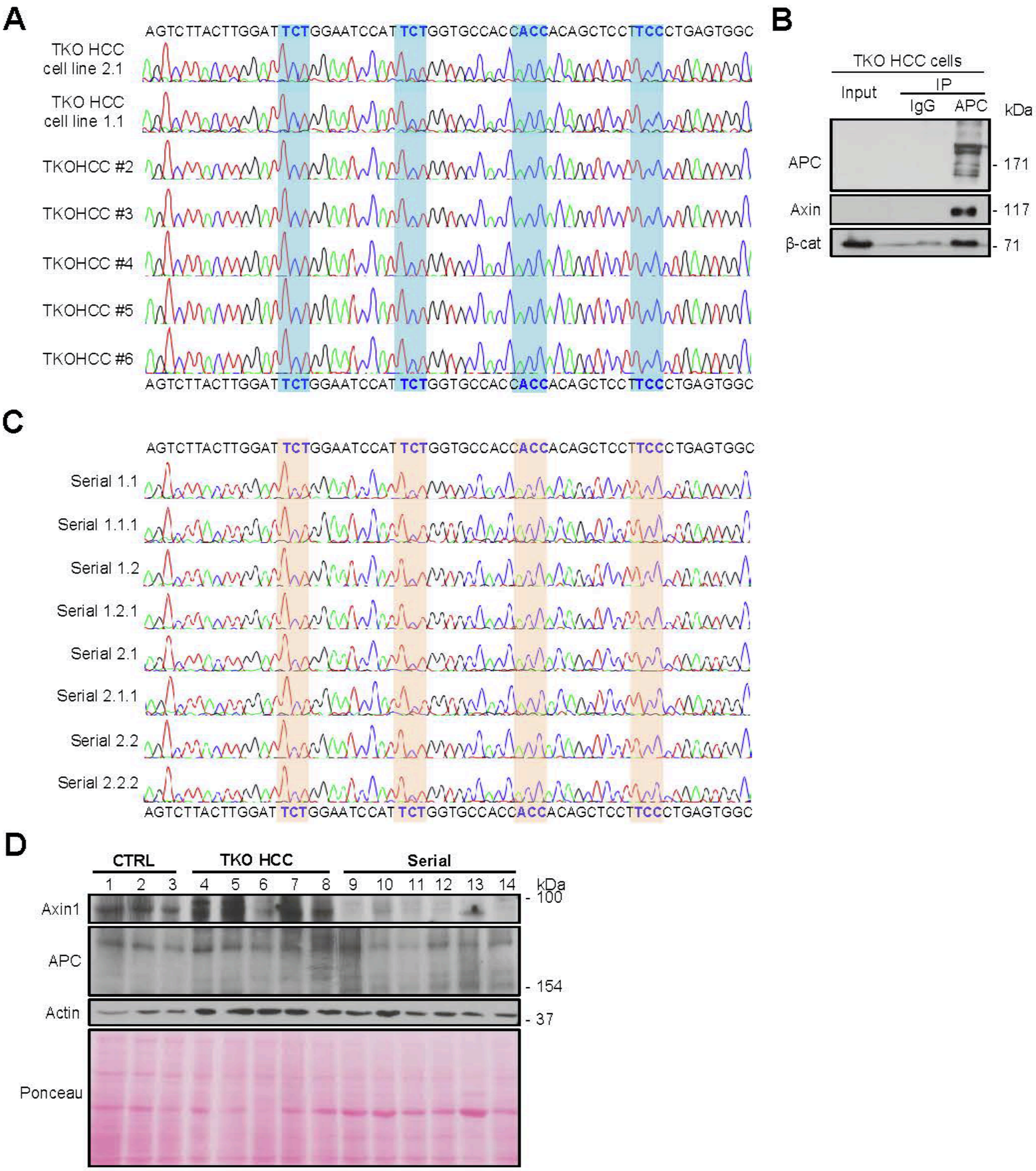

Supplementary Figure 3. Wnt/β-catenin pathway status in TKO HCC and serially transplanted tumors

A *ctnnb1* cDNA was sequenced in both TKO HCC derived cell lines (2.1 and 1.1) as well as in 5 additional independent primary TKO HCC tumors to determine the presence of mutations in the serine/threonine residues targeted by the destruction complex (highlighted in blue). Note: the entire coding sequence was analyzed in this process without any evidence of mutations in *ctnnb1* coding sequence. **B** IP for IgG and APC in TKO HCC cells. The presence of APC, Axin, and β-catenin in the pull-down

fraction was determined by immunoblot. **C** *ctnnb1* cDNA was sequenced in serially transplanted tumors. Regions highlighted in orange correspond to the phosphorylation sites. **D** Immunoblot of Axin1 and APC in control (CTRL) livers (n=3), primary TKO HCC tumors (n=5) and serially transplanted TKO HCC (Serial) tumors (n=6).

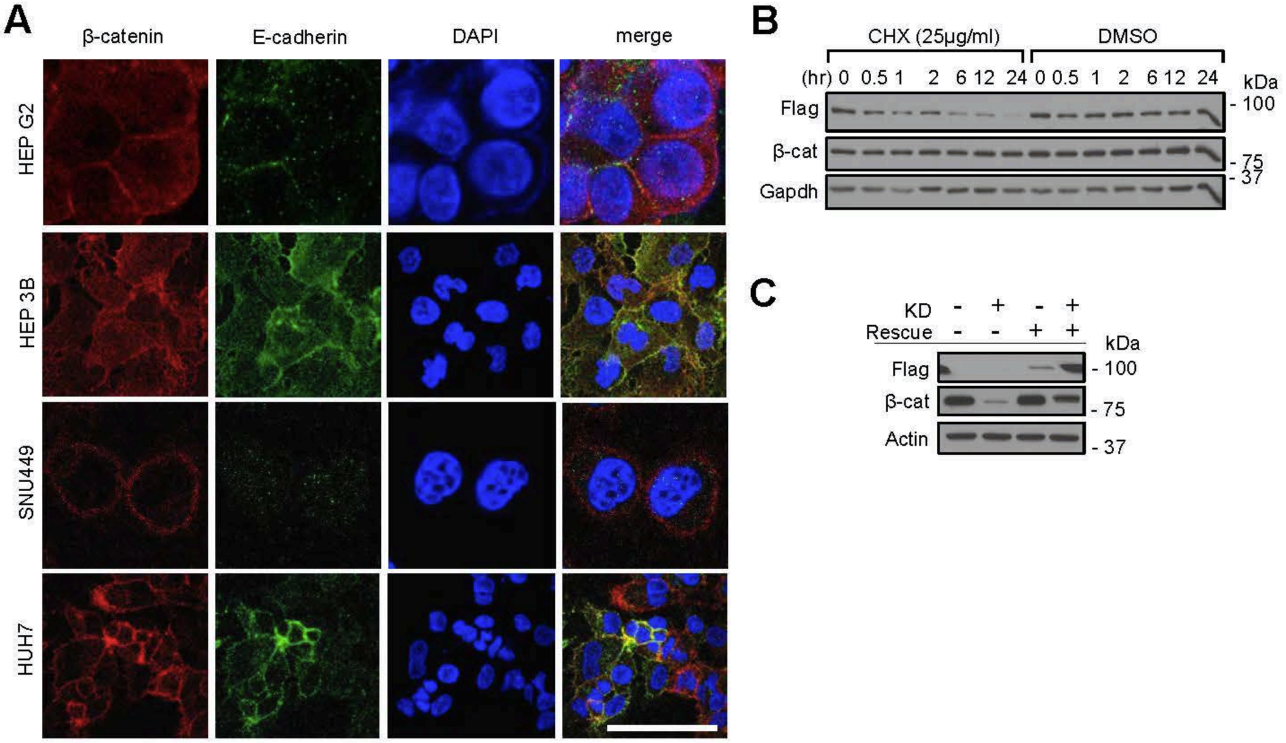

Supplementary Figure 4. Recruitment of β-catenin to the AJ complex

A Representative immunofluorescence staining for β-catenin (red), E-cadherin (green) and DAPI (blue) in human HCC cell lines (HEPG2, HEP3B, SNU449 and HUH7). Scale bar=25µm.**B** Immunoblot for Flag epitope and β-catenin in TKO HCC cells overexpressing Flag-tagged wildtype human β-catenin, treated with 25µg/ml cycloheximide (CHX) for the time indicated (0-24hr). **C** Immunoblot for Flag epitope and β-catenin in TKO HCC cells upon β-catenin KD. (KD=shβcat3; RESCUE=Flag-tagged wildtype human β-catenin).

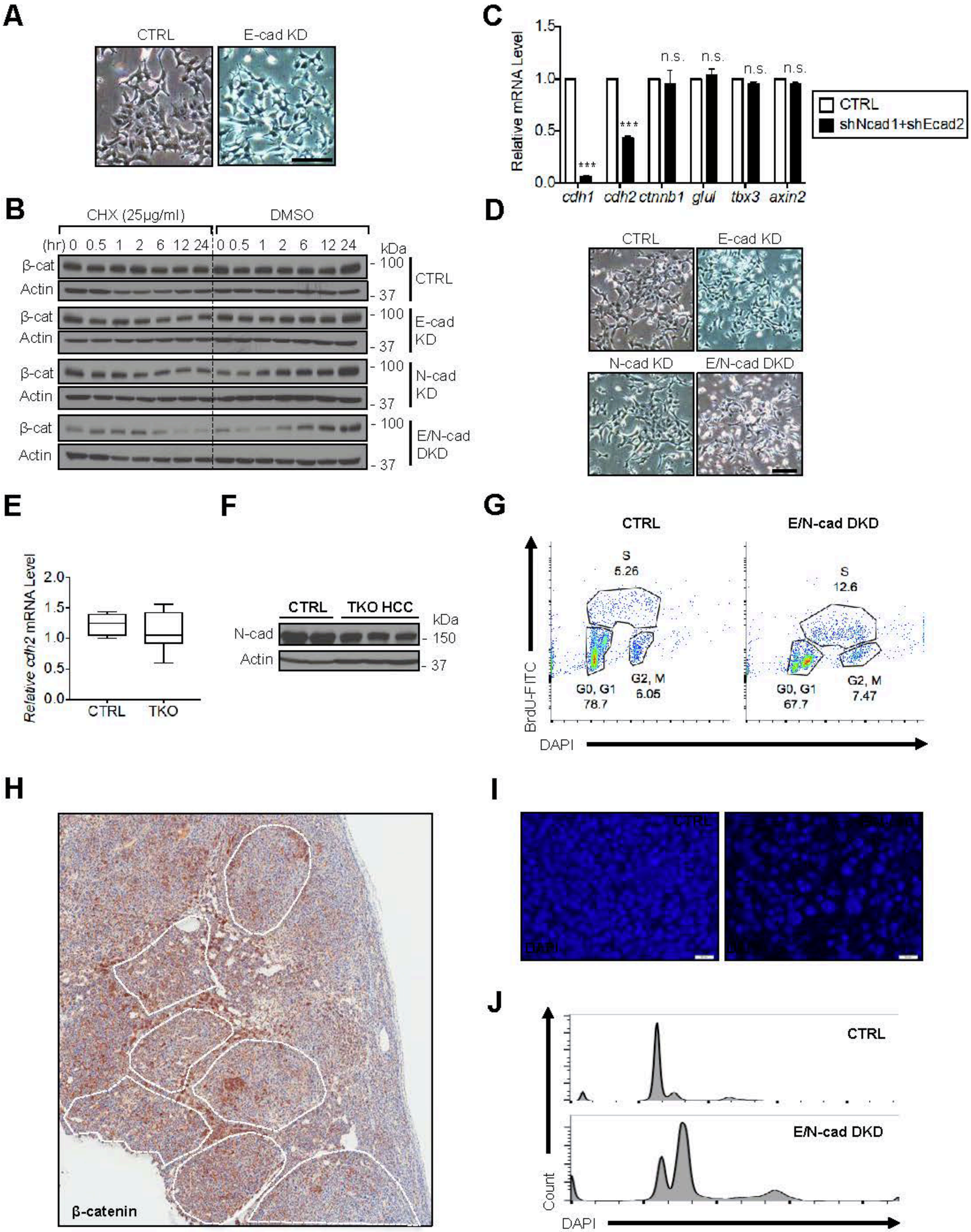
A
CTRL
E-cad KD
B
CHX (25μg/ml)
DMSO
(hr) 0 0.5 1 2 6 12 24 0 0.5 1 2 6 12 24
kDa
β-cat
Actin
- 100
- 37
CTRL
E-cad KD
N-cad KD
E/N-cad DKD
C
Relative mRNA Level
1.5
1.0
0.5
0.0
***
n.s.
CTRL
shNcad1+shEcad2
cdh1 cdh2 ctnnb1 glul tbx3 axin2
D
CTRL
E-cad KD
N-cad KD
E/N-cad DKD
E
Relative cdh2 mRNA Level
2.0
1.5
1.0
0.5
0.0
CTRL
TKO
F
CTRL
TKO HCC
kDa
N-cad
Actin
- 150
- 37
G
CTRL
E/N-cad DKD
S
5.26
G0, G1
78.7
G2, M
6.05
S
12.6
G0, G1
67.7
G2, M
7.47
BrdU-FITC
DAPI
H
β-catenin
I
CTRL
DAPI
J
CTRL
E/N-cad DKD
Count
DAPI

Supplementary Figure 5. In vitro and in vivo consequences of E/N-cadherin DKD in TKO HCC cells

A Representative phase contrast images of TKO HCC cells expressing control (CTRL) or shEcad2 for E-cadherin KD. Scale bar=250μm. **B** Immunoblot for β-catenin in TKO HCC cells expressing an empty vector as a control (CTRL), shEcad2 for E-cadherin KD, shNcad1 for N-cadherin KD, or a combination of both for E/N-cadherin DKD. Cells were treated with 25μg/ml cycloheximide(CHX) for the time indicated (0- 24hr). **C** RT-qPCR analysis for *cdh1*, *cdh2*, *ctnnb1*, and select Wnt target gene mRNA levels (*glul*, *tbx3*, *axin2*) in TKO HCC cells with E/N-cadherin DKD (n=2). **D** Representative phase contrast images of TKO HCC cells expressing empty vector control (CTRL) or shEcad2 for E-cadherin KD, shNcad1 for N-cadherin KD, or shEcad2+shNcad1 for E/N-cadherin DKD. Scale bar=250μm. **E** RT-qPCR analysis for cdh2 mRNA levels in CTRL (n=4) livers and TKO HCC (n=10). CTRL: 1.236 ± 0.09087 with lowest value=1 and highest value=1.433. TKO HCC: 1.116 ± 0.09648 with lowest value=0.596 and highest value=1.563. **F** N-cadherin expression in control (CTRL) livers (n=2) and primary TKO HCC (n=3), as determined by immunoblot. **G** Representative BrdU/DAPI FACS plots of tumor cells from control subcutaneous tumor (CTRL) and E/N-cadherin DKD subcutaneous tumor (E/N-cad DKD). **H** Representative immunohistochemistry (low-magnification) for β-catenin in a subcutaneous tumor from TKO HCC cells with E/N-cadherin DKD. Independent clones are delineated by white dotted lines. Note the presence of β-catenin expressing cell clusters at the margin of these independent clones. **I** Representative DAPI staining of tumors from control (CTRL) or upon E/N-cadherin DKD. Scale bar=20μm. **J** Representative ploidy analysis (DAPI) of tumor cells from control subcutaneous tumor (CTRL) and E/N-cadherin DKD subcutaneous tumor (E/N-cad DKD). Data are represented as mean ±SEM. *$p < 0.05$, **$p < 0.01$, and ***$p < 0.001$.

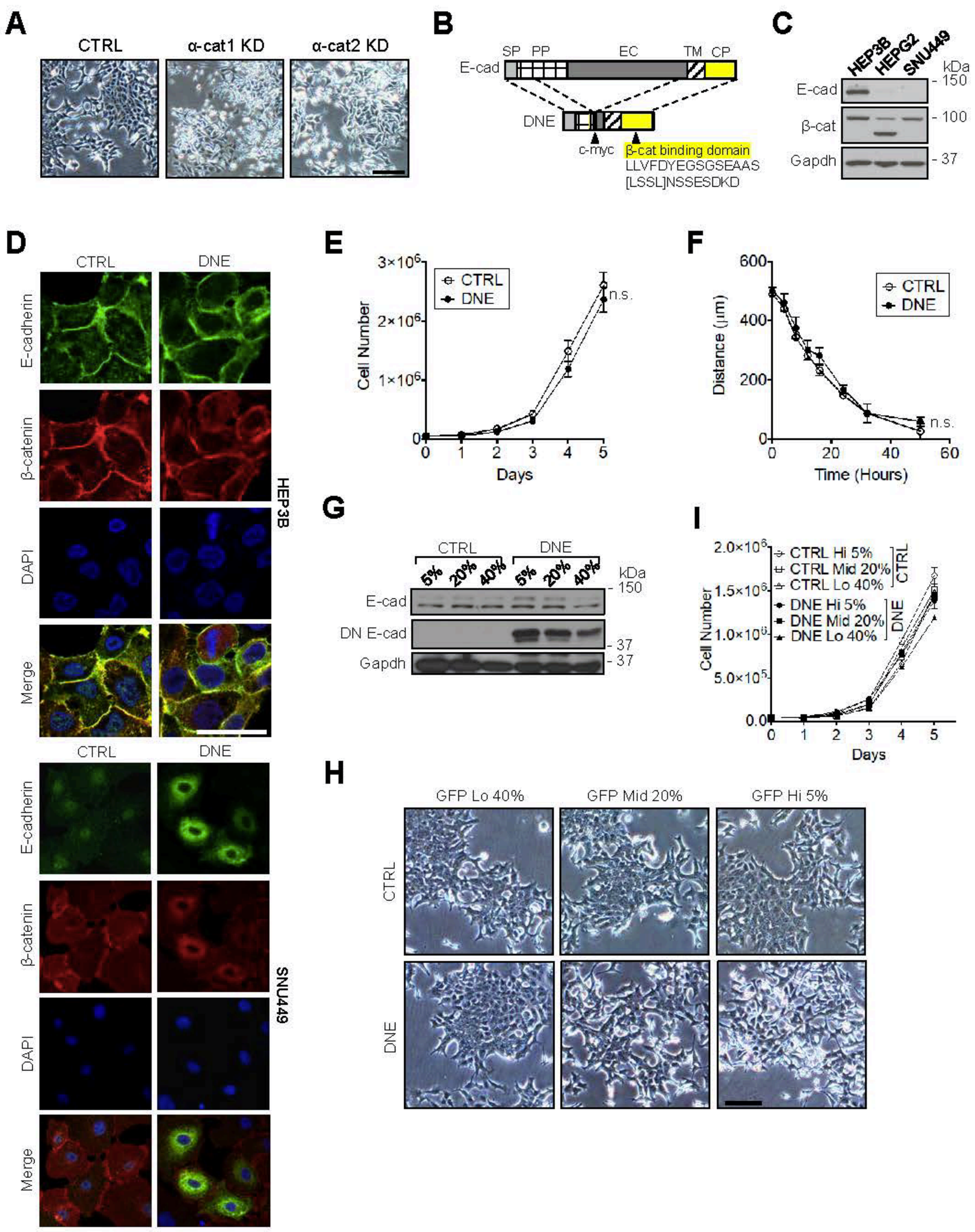

Supplementary Figure 6. Cytoskeleton maintenance and cell-cell interaction do not promote HCC cell survival

A Representative phase contrast images of TKO HCC cells expressing control (CTRL) or shαcat1-2 for α-catenin KD. Scale bar=250μm. **B** The structure of dominant negative E-cadherin (DN E-cadherin). **C** Immunoblot for E-cadherin and β-catenin

in human HCC cell lines (HEP3B, HEPG2, SNU449). **D** IF for E-cadherin (green), β-catenin (red) and DAPI (blue) in HEP3B and SNUC449 cells expressing control (CTRL) or DNE. Scale bar=25μm. **E** Growth curve of TKO HCC cells expressing control (CTRL) or DNE (n=3). **F** Wound healing assay performed on TKO HCC cells expressing control (CTRL) or DNE (n=3). The distance between the two extremities of the wound was measured over the course of 50 hours. **G** Immunoblot for E-cadherin and DN E-cadherin in different fractions of TKO HCC cells infected with empty MigR1 (CTRL) or MigR1 DNE (DNE). Three cellular fractions of each population were isolated by flow cytometry based on the GFP expression: percentile 95 to 100% (5%), percentile 75 to 95% (20%) and percentile 35 to 75% (40%). **H** Representative phase contrast images of TKO HCC cells expressing control (CTRL) or DNE, fractioned based on GFP expression. Scale bar=250μm. **I** Growth curve of TKO HCC cells from the 5%, 20%, 40% groups infected with either CTRL or DNE. Data are represented as mean ±SEM. *p < 0.05, **p < 0.01, and ***p < 0.001.

A

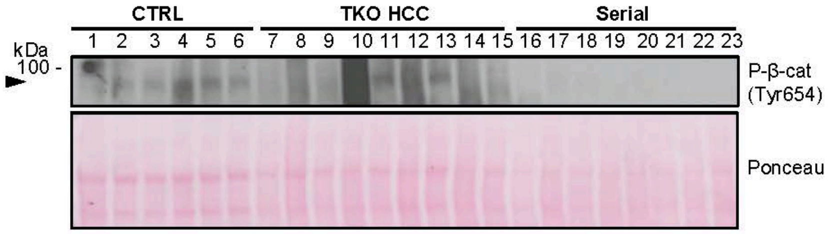

B

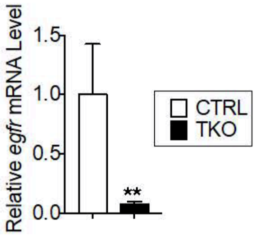

Supplementary Figure 7. Phosphoregulation of β-catenin may regulate AJ complex stability

A Expressions of P-β-catenin(Tyr654) was detected in control livers (1-6), primary TKO HCC tumors (7-15), and serially transplanted TKO HCC (Serial) tumors (16-23) by immunoblot. Ponceau serves as loading control. **B** RT-qPCR analysis for *egfr* mRNA levels in control livers (n=4) and TKO HCC tumors (n=9). Data are represented as mean ±SEM. $*p < 0.05$, $**p < 0.01$, and $***p < 0.001$.

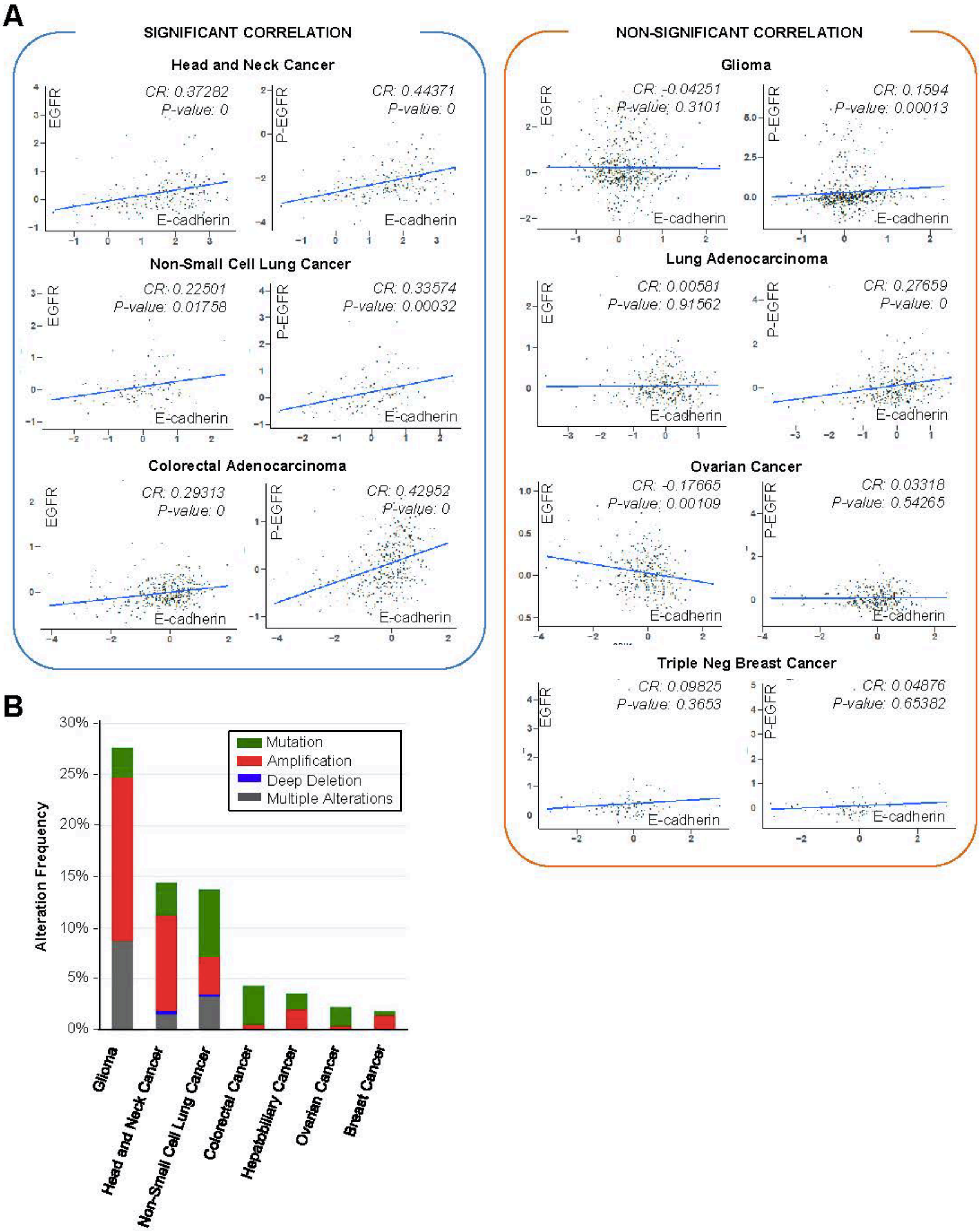
A
SIGNIFICANT CORRELATION
Head and Neck Cancer
EGFR
CR: 0.37282
P-value: 0
E-cadherin
P-EGFR
CR: 0.44371
P-value: 0
E-cadherin
Non-Small Cell Lung Cancer
EGFR
CR: 0.22501
P-value: 0.01758
E-cadherin
P-EGFR
CR: 0.33574
P-value: 0.00032
E-cadherin
Colorectal Adenocarcinoma
EGFR
CR: 0.29313
P-value: 0
E-cadherin
P-EGFR
CR: 0.42952
P-value: 0
E-cadherin
NON-SIGNIFICANT CORRELATION
Glioma
EGFR
CR: -0.04251
P-value: 0.3101
E-cadherin
P-EGFR
CR: 0.1594
P-value: 0.00013
E-cadherin
Lung Adenocarcinoma
EGFR
CR: 0.00581
P-value: 0.91562
E-cadherin
P-EGFR
CR: 0.27659
P-value: 0
E-cadherin
Ovarian Cancer
EGFR
CR: -0.17665
P-value: 0.00109
E-cadherin
P-EGFR
CR: 0.03318
P-value: 0.54265
E-cadherin
Triple Neg Breast Cancer
EGFR
CR: 0.09825
P-value: 0.3653
E-cadherin
P-EGFR
CR: 0.04876
P-value: 0.65382
E-cadherin
B
Alteration Frequency
30%
25%
20%
15%
10%
5%
0%
Mutation
Amplification
Deep Deletion
Multiple Alterations
Glioma
Head and Neck Cancer
Non-Small Cell Lung Cancer
Colorectal Cancer
Hepatobiliary Cancer
Ovarian Cancer
Breast Cancer

Supplementary Figure 8. EGFR/P-EGFR/E-cadherin expressions in various human epithelial cancers

A Correlation between EGFR/P-EGFR(Tyr1068) and E-cadherin in select epithelial cancers. The group on the left (head & neck cancer, non-small cell lung cancer (NSCLC), and colorectal adenocarcinoma) displays significant correlation between EGFR and E-cadherin expression. The group on the right (glioma, lung adenocarcinoma, , ovarian cancer, and triple negative breast cancer) displays non-significant correlation. (CR=correlation ratio). **B** Alteration frequency in the following epithelial cancers: glioma, head & neck cancer, non-small cell lung cancer (NSCLC), colorectal adenocarcinoma, hepatobiliary cancer, ovarian cancer, and breast cancer.

Supplementary Figure 9. Uncropped immunoblots

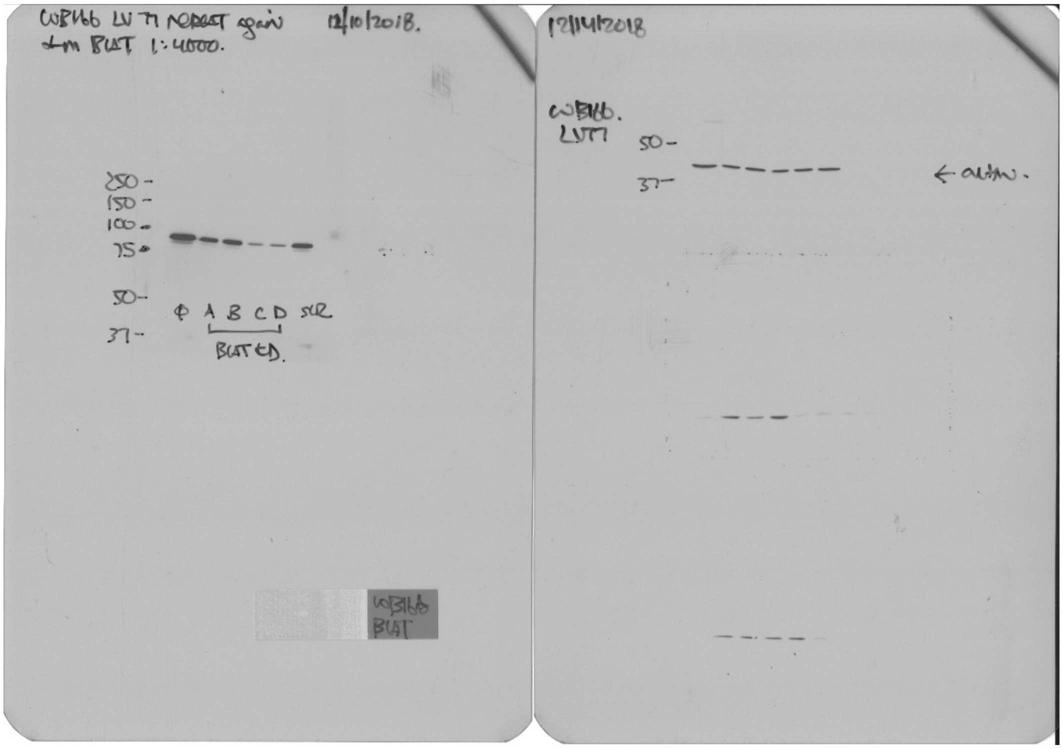

Figure 2(F)

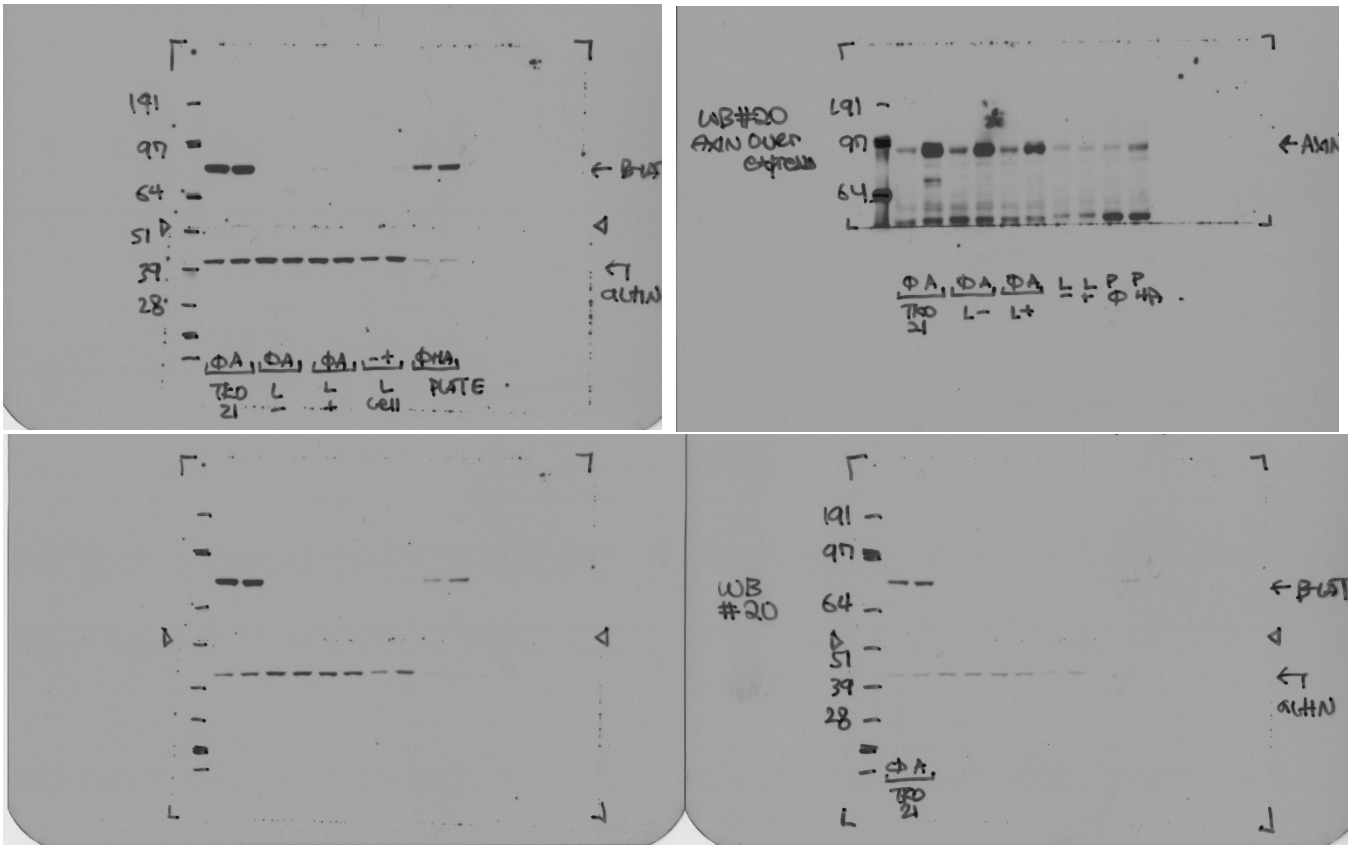

Figure 3(E)

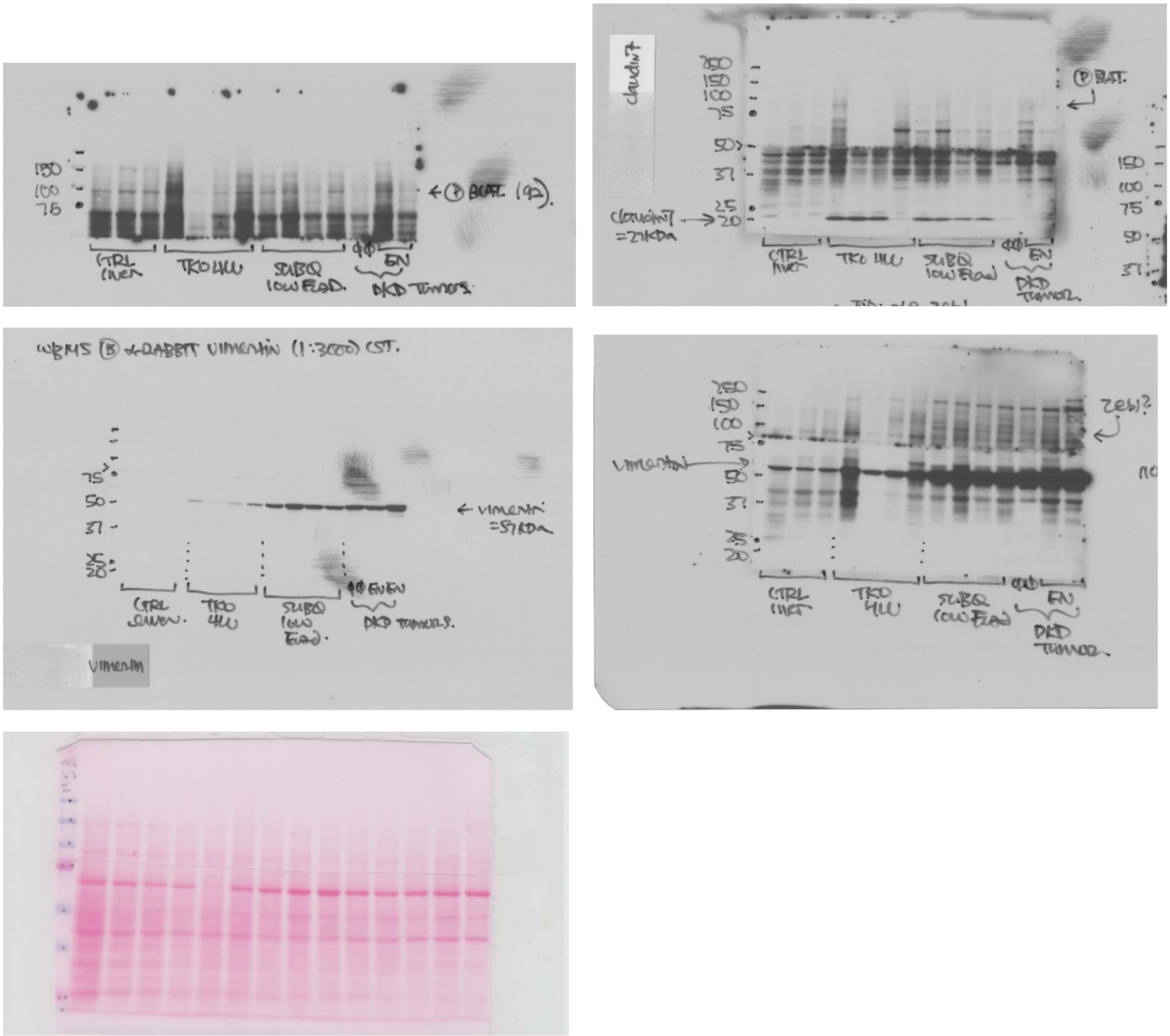
CTRL liver
TKO 4W
SUBQ low Ezad
DKD tumors
claudin7
vimentin
=57kDa
Zeb1?

Figure 3(N)

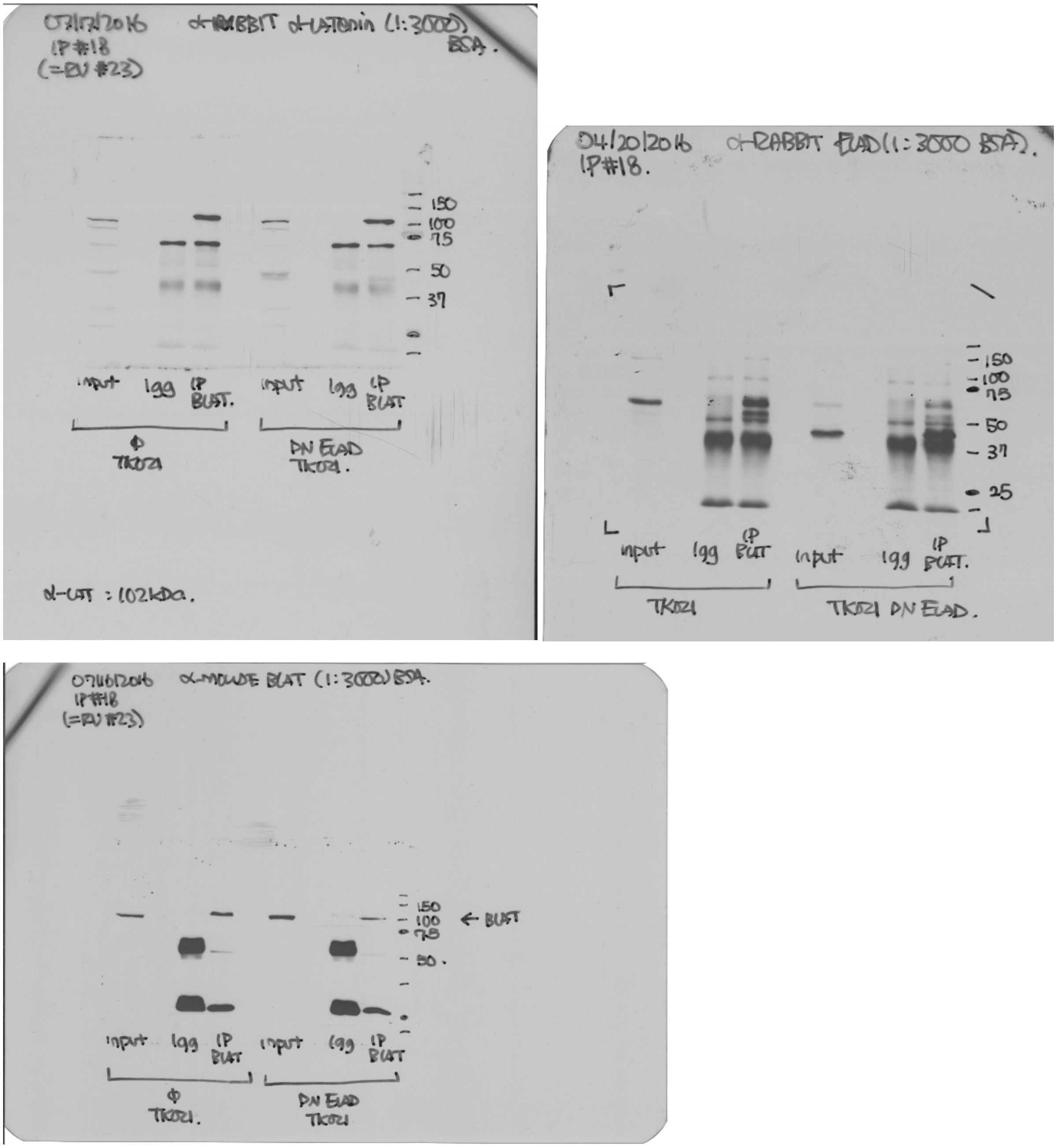
07/17/2016 α-RABBIT α-LATEDIN (1:3000) BSA.
IP #18
(=RV #23)
150
100
75
50
37
input Igg IP BLAT. input Igg IP BLAT
Φ TK021
DN ELAD TK021.
α-LAT : 102kDa.
04/20/2016 α-RABBIT ELAD (1:3000 BSA).
IP#18.
150
100
75
50
37
25
input Igg IP BLAT input Igg IP BLAT.
TK021
TK021 DN ELAD.
07/16/2016 α-MOUSE BLAT (1:3000) BSA.
IP#18
(=RV #23)
150
100
75
50.
← BLAT
input Igg IP BLAT input Igg IP BLAT
Φ TK021.
DN ELAD TK021

Figure 4(B)

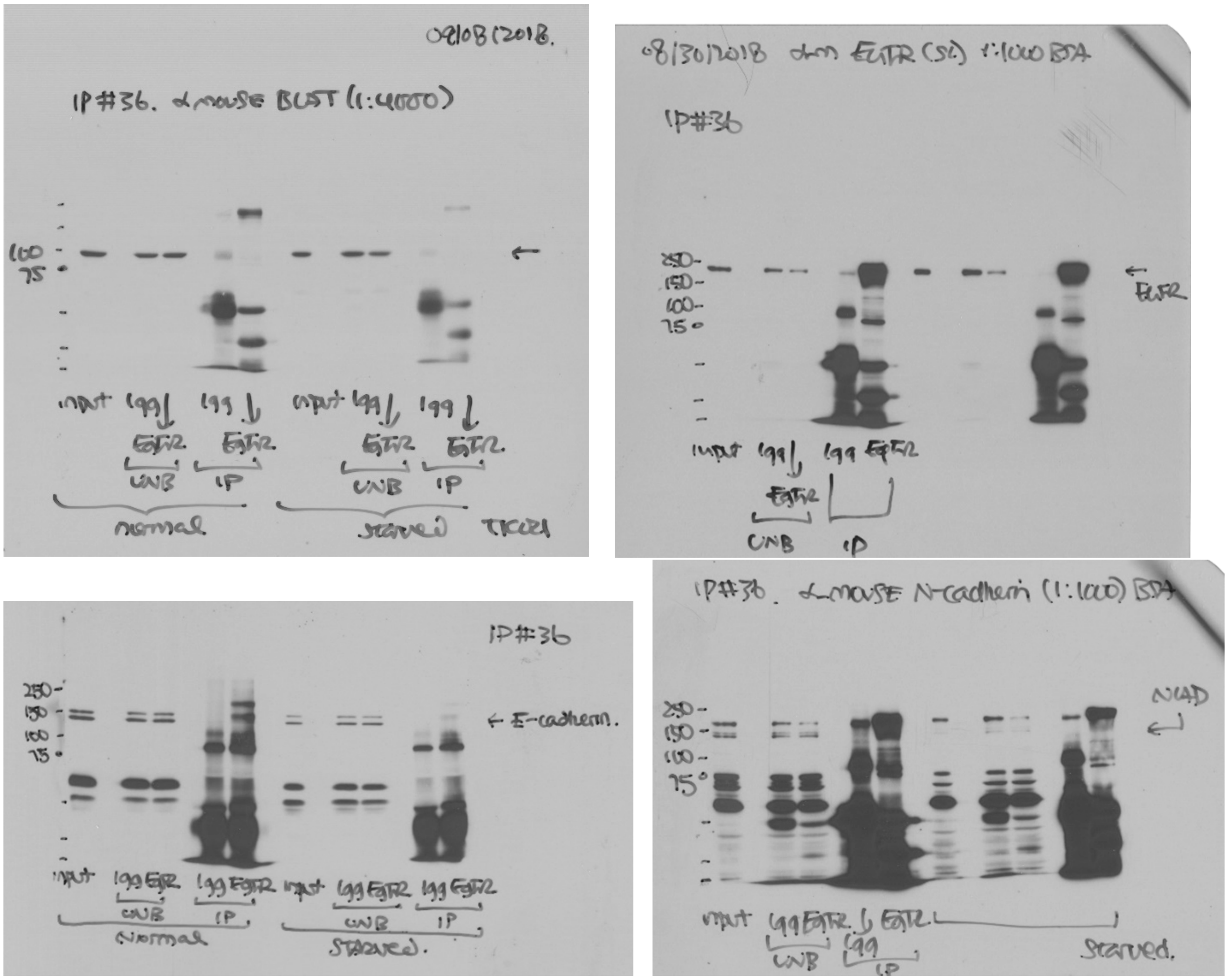

Figure 7(A)

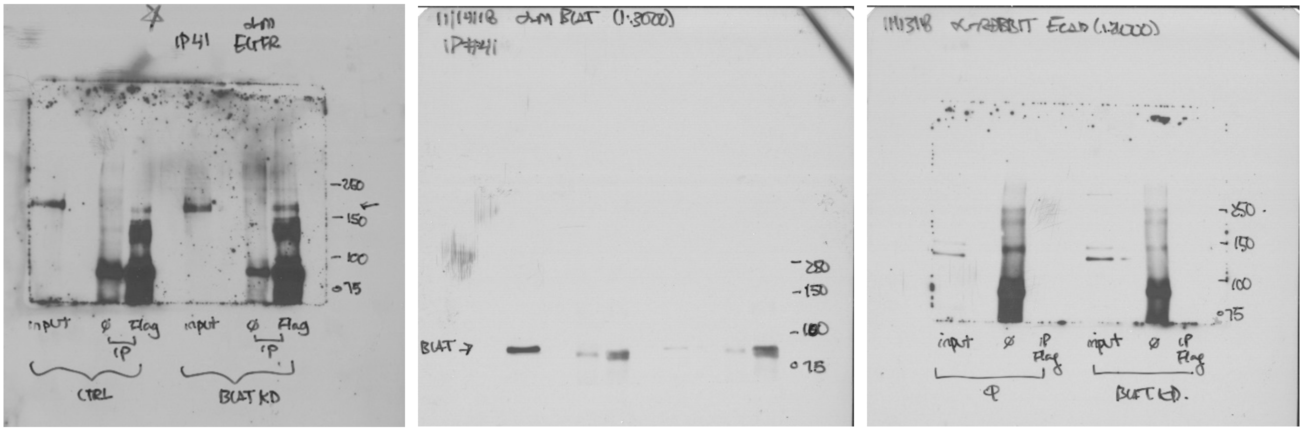

Figure 7(I)

Supplementary Table 1. Mass Spectrometry Analysis Summary

Genes	1679 Proteins in 1167 Clusters (16 Decoys and 1 Hidden)	MW	TKO21 CTRL	TKO21 CTRL	TKO21 shEcad2:	Keyword
			IP: IgG	IP: β-cat	IP: β-cat	
**Cdh1**	Cadherin-1 OS=Mus musculus GN=Cdh1 PE=1 SV=1	98 kDa		222	15	Cell junction
**Cdh2**	Cadherin-2 OS=Mus musculus GN=Cdh2 PE=1 SV=2	100 kDa		215	273	Cell junction
**APC**	Cluster of Adenomatosis polyposis coli OS=Mus musculus GN=Apc PE=1 SV=1 (B2RUG9)	311 kDa		126	101	Adhesion/Wnt
**Cdh6**	Cluster of Cadherin-6 OS=Mus musculus GN=Cdh6 PE=1 SV=2 (P97326)	88 kDa		123	116	Cell junction
**Arvcf**	Cluster of Armadillo repeat protein deleted in velo-cardio-facial syndrome homolog OS=Mus musculus GN=Arvcf PE=1 SV=2 (P98203)	105 kDa		118	72	Cell junction
**Krt77**	Keratin 77 OS=Mus musculus GN=Krt77 PE=2 SV=1	61 kDa		60	32	Cell structure
**Anxa1**	Annexin OS=Mus musculus GN=Anxa1 PE=2 SV=1	39 kDa		15	13	Membrane protein; anti-inflammatory
**Ubr1**	E3 ubiquitin-protein ligase UBR1 OS=Mus musculus GN=Ubr1 PE=1 SV=2	200 kDa		12	3	Ubiquitylation
**Lrrc59**	Putative uncharacterized protein OS=Mus musculus GN=Lrrc59 PE=2 SV=1	20 kDa	2	6	16	Nuclear import
**Cct3**	T-complex protein 1 subunit gamma OS=Mus musculus GN=Cct3 PE=2 SV=1	61 kDa	2	8	5	Protein folding
**Pkp4**	Cluster of Putative uncharacterized protein (Fragment) OS=Mus musculus GN=Pkp4 PE=2 SV=1 (Q3UIX3)	88 kDa	3	101	69	Cell junction
**Aldoa**	Cluster of Fructose-bisphosphate aldolase OS=Mus musculus GN=Aldoa PE=1 SV=1 (A6ZI44)	45 kDa	3	10	9	Glycolysis (liver, kidney, intestines)
**Xpnpep3**	Probable Xaa-Pro aminopeptidase 3 OS=Mus musculus GN=Xpnpep3 PE=2 SV=1	57 kDa	3	14	19	Mitochondrial
**Tcp1**	T-complex protein 1 subunit alpha OS=Mus musculus GN=Tcp1 PE=1 SV=3	60 kDa	4	10	8	Protein folding
**Acot9**	Cluster of Acyl-coenzyme A thioesterase 9, mitochondrial OS=Mus musculus GN=Acot9 PE=1 SV=1 (Q9R0X4)	51 kDa	6	13	12	n/a
**Pcmt1**	Protein-L-isoaspartate O-methyltransferase OS=Mus musculus GN=Pcmt1 PE=1 SV=1	30 kDa	7	20	25	Protein repair
**Tfg**	Cluster of Putative uncharacterized protein OS=Mus musculus GN=Tfg PE=2 SV=1 (Q9D828)	42 kDa	8	135	296	Protein folding
**Ubr2**	Putative uncharacterized protein OS=Mus musculus GN=Ubr2 PE=2 SV=1	199 kDa	8	46	10	Ubiquitylation
**Ctnnd1**	Cluster of Isoform 2 of Catenin delta-1 OS=Mus musculus GN=Ctnnd1 (P30999-2)	102 kDa	10	56	23	Cell adhesion
**Ctnnb1**	Cluster of Catenin beta-1 OS=Mus musculus GN=Ctnnb1 PE=1 SV=1 (Q02248)	85 kDa	13	1013	789	Cell adhesion
**Jup**	Junction plakoglobin OS=Mus musculus GN=Jup PE=1 SV=3	82 kDa	14	117	80	Cell adhesion
**Ubr3**	Cluster of E3 ubiquitin-protein ligase UBR3 OS=Mus musculus GN=Ubr3 PE=1 SV=3 (Q5U430)	213 kDa	18	36	20	Ubiquitylation
**Naa50**	Isoform 2 of N-alpha-acetyltransferase 50 OS=Mus musculus GN=Naa50	19 kDa	21	36	47	Nuclear
**Ctnna1**	Cluster of Putative uncharacterized protein OS=Mus musculus GN=Ctnna1 PE=2 SV=1 (Q3TGQ3)	100 kDa	28	1606	1275	Cell adhesion
**Itgav**	Cluster of Integrin alpha-V OS=Mus musculus GN=Itgav PE=1 SV=2 (P43406)	115 kDa	28	54	46	Cell adhesion

Supplementary Table 2. qPCR Primers List

Type	Primer Name		Primer Sequence	Target Species
**qPCR**	*Axin2*	F	TTC AAG GAG CAG CTC AGC AA	Mouse
		R	CAG GCA AAT TCG TCA CTC GC	Mouse
	Cdh1	F	AGG AAA TGC ACC CCT CCA AT	Mouse
		R	AAT CGG CCA GCA TTT TCT G	Mouse
	Cdh2	F	AGG ATG TGC ACG AAG GAC AG	Mouse
		R	CTT GAA ATC TGC TGG CTC GC	Mouse
	Ctnnb1	F	CCT TGG ATA TCG CCA GGA T	Mouse
		R	TGG CCG TAT CCA CCA GAG	Mouse
	EGFR	F	TGC CAC CTA TGC CAC GCC AAC	Mouse
		R	TGA GAC CTC TGG CTG GCC CA	Mouse
	Gapdh	F	TTC ACC ACC ATG GAG AAG GC	Mouse
		R	CCC TTT TGG CTC CAC CCT	Mouse
	Glul	F	CAG GGA CAT CGT GGA GGC TC	Mouse
		R	TTG GAA TTC CCA CTG GGC AG	Mouse
	Lgr5	F	CAG GCC GTC TGT GAT CAG TT	Mouse
		R	GCA GCC TGA CAA ACT GGG TA	Mouse
	Tbx3	F	AGC GAT CAC GCA ACG TGG CA	Mouse
		R	GGC TTC GCT GGG ACA CAG ATC TTT	Mouse

Supplementary Table 3. PCR Amplification and Sequencing Primers List

Type	Primer Name		Primer Sequence	Target Region
**Amplification**	β-catenin (delaCoste)	F1	GCG TGG ACA ATG GCT ACT CAA G	*mouse ctnnb1* E2 (ATG)
	β-catenin (delaCoste)	R1	TAT TAA CTA CCA CCT GGT CCT C	*mouse ctnnb1* E5
	β-catenin (delaCoste)	F2	ACG CGG AAC TTG CCA CAC GTG C	*mouse ctnnb1* E4
	β-catenin (delaCoste)	R2	TTC AGC ACT CTG CTT GTG GTC	*mouse ctnnb1* E7
	β-catenin cDNA PCR	R	GCC TAA AAC CAT TCC CAC CCT A	*mouse ctnnb1* E15 (TAA)
**Sequencing**	β-catenin cDNA seq	F1	GAC GCT GCT CAT CCC ACT AA	*mouse ctnnb1* E4-E6
	β-catenin cDNA seq	F2	ACT GTT CTA CGC CAT CAC GA	*mouse ctnnb1* E6-E8
	β-catenin cDNA seq	F3	TCA GAT GCA GCG ACT AAG CA	*mouse ctnnb1* E8-E11
	β-catenin cDNA seq	F4	CAG CAA ATC ATG CGC CTT TG	*mouse ctnnb1* E10-E14
	β-catenin cDNA seq	F5	CAG GAC AAG AGG CCA TTG AA	*mouse ctnnb1* E13-E15
	β-catenin exon5	R	GCT GGG ATG CCA CCA GAC TTA A	*mouse ctnnb1* E5
