# Supplementary material for: Promotion of growth factor signaling as a critical function of β-catenin during HCC progression
Source: Nat Commun. 2019 Apr 23;10:1909. doi: 10.1038/s41467-019-09780-z (PMC6478918; doi:10.1038/s41467-019-09780-z)
Supplement: Supplementary file 3 — Reporting Summary [file 41467_2019_9780_MOESM3_ESM.pdf]

nature research

Corresponding author(s): *Double-blind peer review submissions: write DBPR and your manuscript number here instead of author names.*

Last updated by author(s): *YYYY-MM-DD*

Reporting Summary

Nature Research wishes to improve the reproducibility of the work that we publish. This form provides structure for consistency and transparency in reporting. For further information on Nature Research policies, see Authors & Referees and the Editorial Policy Checklist.

Statistics

For all statistical analyses, confirm that the following items are present in the figure legend, table legend, main text, or Methods section.

n/a	Confirmed	
☐	☒	The exact sample size (*n*) for each experimental group/condition, given as a discrete number and unit of measurement
☒	☐	A statement on whether measurements were taken from distinct samples or whether the same sample was measured repeatedly
☐	☒	The statistical test(s) used AND whether they are one- or two-sided<br>*Only common tests should be described solely by name; describe more complex techniques in the Methods section.*
☒	☐	A description of all covariates tested
☒	☐	A description of any assumptions or corrections, such as tests of normality and adjustment for multiple comparisons
☒	☐	A full description of the statistical parameters including central tendency (e.g. means) or other basic estimates (e.g. regression coefficient) AND variation (e.g. standard deviation) or associated estimates of uncertainty (e.g. confidence intervals)
☒	☐	For null hypothesis testing, the test statistic (e.g. *F*, *t*, *r*) with confidence intervals, effect sizes, degrees of freedom and *P* value noted<br>*Give P values as exact values whenever suitable.*
☒	☐	For Bayesian analysis, information on the choice of priors and Markov chain Monte Carlo settings
☒	☐	For hierarchical and complex designs, identification of the appropriate level for tests and full reporting of outcomes
☒	☐	Estimates of effect sizes (e.g. Cohen's *d*, Pearson's *r*), indicating how they were calculated

Our web collection on statistics for biologists contains articles on many of the points above.

Software and code

Policy information about availability of computer code

Data collection	No software was used
Data analysis	Microsoft Excel, Scaffold,

For manuscripts utilizing custom algorithms or software that are central to the research but not yet described in published literature, software must be made available to editors/reviewers. We strongly encourage code deposition in a community repository (e.g. GitHub). See the Nature Research guidelines for submitting code & software for further information.

Data

Policy information about availability of data

All manuscripts must include a data availability statement. This statement should provide the following information, where applicable:

- Accession codes, unique identifiers, or web links for publicly available datasets
- A list of figures that have associated raw data
- A description of any restrictions on data availability

Provide your data availability statement here.

Field-specific reporting

Please select the one below that is the best fit for your research. If you are not sure, read the appropriate sections before making your selection.

☒ Life sciences ☐ Behavioural & social sciences ☐ Ecological, evolutionary & environmental sciences

For a reference copy of the document with all sections, see nature.com/documents/nr-reporting-summary-flat.pdf

Life sciences study design

All studies must disclose on these points even when the disclosure is negative.

Sample size	Experiments are always performed with a minimum of three independent samples per conditions AND/OR are performed a minimum of three independent times, at least in duplicates. Statistical significance is achieved by including the sample size in the parameters.
Data exclusions	No data were excluded for analysis. Outliers were identified in Figure 8B. Statistical tests were used (and described accordingly) to identify their outlier status.
Replication	All experiments are performed a minimum of three independent times, in duplicates when possible (such as cell growth assay). No replicates were excluded during the analysis of the data
Randomization	No randomization procedures were performed in this mechanistic study
Blinding	For the analysis of tumor array (figure1), the board certified pathologist was not made aware of the identity of the samples present on the array during her evaluation of staining intensity and localization. Other assays did not involve blinding procedures.

Reporting for specific materials, systems and methods

We require information from authors about some types of materials, experimental systems and methods used in many studies. Here, indicate whether each material, system or method listed is relevant to your study. If you are not sure if a list item applies to your research, read the appropriate section before selecting a response.

Materials & experimental systems

n/a	Involved in the study	
☐	☒	Antibodies
☐	☒	Eukaryotic cell lines
☒	☐	Palaeontology
☐	☒	Animals and other organisms
☒	☐	Human research participants
☒	☐	Clinical data

Methods

n/a	Involved in the study	
☒	☐	ChIP-seq
☐	☒	Flow cytometry
☒	☐	MRI-based neuroimaging

Antibodies

Antibodies used	All antibodies are commercial. The reference and dilution is described in the Methods section
Validation	A negative control was included in the experimental plan whenever possible.

Eukaryotic cell lines

Policy information about cell lines

Cell line source(s)	TKO HCC cell lines are derived from individual primary tumors and their origin is described in the Methods section. Other cell lines used were obtain from commercial sources, as identified in the methods section of the manuscript.
Authentication	None of the commercial cell lines were authenticated. TKO HCC cell lines were previously authenticated (upon their derivation-Viatour et all JEM 2011) for Rb family protein expression.
Mycoplasma contamination	Cell lines were not tested for mycoplasma contamination
Commonly misidentified lines (See ICLAC register)	N/A

Animals and other organisms

Policy information about studies involving animals; ARRIVE guidelines recommended for reporting animal research

Laboratory animals	Rb family triple knock out mice, mix 129/Bl6 of both genders, used between two and 6 months of age. Nod/scid mice (two to six months old) were obtained from Charles Rivers
Wild animals	The study did not involve wild type animals
Field-collected samples	The study did not involve field collected samples

Ethics oversight	Department of Veterinary Resources at the Children's Hospital of Philadelphia; IACUC

Note that full information on the approval of the study protocol must also be provided in the manuscript.

Flow Cytometry

Plots

Confirm that:

- [x] The axis labels state the marker and fluorochrome used (e.g. CD4-FITC).
- [x] The axis scales are clearly visible. Include numbers along axes only for bottom left plot of group (a 'group' is an analysis of identical markers).
- [x] All plots are contour plots with outliers or pseudocolor plots.
- [x] A numerical value for number of cells or percentage (with statistics) is provided.

Methodology

Sample preparation	Cultured cells were collected by trypsin treatment and single cell suspension were processed for staining.
Instrument	BD Fortessa for analysis and BD Facs Aria for sorting
Software	Flow Jo for data analysis
Cell population abundance	Post sort were randomly performed based on machine availability. Post sort analysis always revealed an efficiency superior to 90%. The vast majority of the 10% populations were dimer cells, which is a frequent phenomenon during post sort analysis
Gating strategy	Cells are gated on FSC/SSC and A/H for doublets exclusion.

- [ ] Tick this box to confirm that a figure exemplifying the gating strategy is provided in the Supplementary Information.
